# The Impact of Periodontal Therapy on Disease Activity in Patients with Rheumatoid Arthritis and Concomitant Periodontitis: A Systematic Review and Meta-Analysis

**DOI:** 10.3390/jcm15135099

**Published:** 2026-06-30

**Authors:** Lina Khennoufa, Ana Sofia Vinhas, Josselin Benoit, Rosana Costa, Filomena Salazar, Cristina Cabral, Cátia Reis

**Affiliations:** 1 Faculty of Dental Medicine, University of Health Science, Cooperativa de Ensino Superior Politécnico e Universitário (IUCS-CESPU), 4585-116 Gandra, Portugal; 2 Department of Medicine and Oral Surgery, University of Health Science, Cooperativa de Ensino Superior Politécnico e Universitário (IUCS-CESPU), 4585-116 Gandra, Portugal; 3 Oral Pathology and Rehabilitation Research Unit (UNIPRO), University of Health Science, Cooperativa de Ensino Superior Politécnico e Universitário (IUCS-CESPU), 4585-116 Gandra, Portugal

**Keywords:** periodontitis, periodontal disease, rheumatoid arthritis, assessment of rheumatoid arthritis, periodontal therapy, subgingival instrumentation, supragingival scaling, dental plaque

## Abstract

**Background/Objectives**: The interplay between oral and systemic diseases is highlighted by the shared inflammatory mechanisms and epidemiological associations between periodontitis and rheumatoid arthritis (RA). Building on previous syntheses of the effect of periodontal therapy on RA disease activity, we sought to refine the evidence base through strict restriction to randomized controlled trials (RCTs), separate analysis of the two non-interchangeable formulations of the Disease Activity Score on 28 joints (DAS28-CRP and DAS28-ESR, based on either C-reactive protein or erythrocyte sedimentation rate, respectively), and inclusion of recent randomized trials. We aimed to determine whether the first two steps of periodontal therapy (steps 1 and 2 of the 2020 EFP S3-level clinical practice guideline), delivered through supragingival professional mechanical plaque removal and subgingival instrumentation, reduce DAS28 in adults with concurrent RA and periodontitis. **Methods**: The review protocol was registered in PROSPERO (CRD420261400735). Five databases were searched in accordance with PRISMA 2020. Only RCTs were eligible. Risk of bias was assessed with RoB-2. DAS28-CRP and DAS28-ESR were analyzed in separate random-effects forest plots. Sensitivity analyses addressed adjunctive antibiotics and high baseline disease activity. **Results**: Ten trials (*n* = 430 randomized patients) were included. At 3 months, DAS28-CRP was significantly reduced (between-group MD = −0.84, 95% CI −1.38 to −0.29; change-from-baseline MD = −0.55, −0.92 to −0.19). On DAS28-ESR at 3 months, the change-from-baseline estimate was significant (MD = −1.27, −2.22 to −0.31) and the follow-up estimate concordant in direction but not significant (MD = −0.89, −1.85 to 0.07), with substantial heterogeneity. **Conclusions**: Periodontal therapy may be associated with short-term reductions in RA disease activity, particularly DAS28-CRP at 3 months, with directionally concordant but less certain effects on DAS28-ESR. The evidence remains limited by small sample sizes, risk of bias, substantial heterogeneity of the DAS28-ESR estimates, and sparse follow-up beyond 3 months. As no trial reported individual responder categories, these group-level findings support periodontal therapy as a possible adjunctive measure in RA rather than a predictable, clinically meaningful benefit at the individual patient level.

## 1. Introduction

Periodontitis and rheumatoid arthritis (RA) are two chronic inflammatory conditions whose interconnection has progressively emerged as a paradigm of the interplay between oral and systemic health [1,2]. RA is a systemic autoimmune disease characterized by chronic synovial inflammation, progressive joint destruction, and increased mortality. RA affects approximately 0.5–1.0% of the global population, with a clinical onset that typically occurs after 35 years of age and which has a marked female predominance, with a sex ratio of about 3:1 [3,4]. In European populations, prevalence estimates range from 0.35% to 0.9%, with an incidence four to five times higher in women than in men under 50 years of age, narrowing to approximately 2:1 by the seventh decade [5]. Periodontitis, defined as an inflammatory disease of the tooth-supporting tissues driven by a dysbiotic subgingival biofilm in a susceptible host, is among the most widespread non-communicable diseases worldwide [5]. Severe forms affect approximately 7–11% of the world population and have together been ranked as the sixth most prevalent health condition in the 2010 Global Burden of Disease analysis [5], while milder forms affect close to half of the adult population [5]. Beyond their respective epidemiological burdens, both diseases progressively destroy connective tissue and bone through chronic, cytokine-driven inflammation, share several genetic and environmental risk factors, most notably cigarette smoking, and are increasingly considered manifestations of a common dysregulated inflammatory phenotype [2,5]. More broadly, several rheumatologic conditions are associated with oral and orofacial manifestations, supporting integrated dental and rheumatologic assessment as part of their management.

The clinical, epidemiological, and immunological links between RA and periodontitis are well documented [1,2]. Large observational studies have established that periodontitis occurs more frequently and with greater severity in patients with RA than in the general population, with risk estimates that increase with RA disease activity and severity [5,6]. Importantly, this association does not appear to be a simple consequence of reduced manual dexterity or impaired oral hygiene in RA patients. In a systematic review and meta-analysis of case–control studies, Hussain et al. [7] found no significant effect of RA on periodontal clinical parameters, whereas the converse direction was confirmed: patients with concomitant periodontitis exhibited a higher DAS28 score, with a pooled mean difference of 0.74 points (95% CI 0.25–1.24, *p* < 0.001) and low between-study heterogeneity. At the mechanistic level, both diseases share dysregulated host immune responses, overlapping cytokine networks (TNF-α, IL-1β, IL-6) and activation of the RANKL/OPG axis governing osteoclast genesis, with osteoclastogenic markers reaching their highest serum levels in patients with both conditions, supporting convergence on a common osteoimmunological pathway rather than incidental co-occurrence [8,9].

A microbiological mechanism has been advanced to explain how periodontal infection may contribute to the breach of immune tolerance underlying RA. *Porphyromonas gingivalis*, a keystone periodontal pathogen, is the only known bacterium expressing peptidyl arginine deiminase (PPAD), an enzyme capable of citrullinating host and bacterial proteins to generate neo-antigens that may trigger the production of anti-citrullinated protein antibodies (ACPAs), a hallmark of RA [10,11]. A second pathway implicates *Aggregatibacter actinomycetemcomitans*, whose pore-forming leukotoxin A induces hypercitrullination in host neutrophils and the release of citrullinated cargo through neutrophil extracellular traps, mirroring the citrullination pattern observed in rheumatoid joints [10]. Consistent with this paradigm, antibodies against *p. gingivalis* have been detected before the clinical onset of RA in otherwise healthy individuals, and oral dysbiosis involving *p. gingivalis*, *A. actinomycetemcomitans* and other taxa is observed in first-degree relatives of RA patients and in ACPA-positive individuals at risk of developing the disease [11]. These observations have led some authors to propose that periodontitis may serve as an early indicator of systemic immune dysregulation rather than merely a downstream comorbidity, providing a biological rationale for evaluating whether periodontal therapy modulates RA disease activity [1,11].

Despite this biological rationale, the magnitude, durability, and clinical relevance of the effect of periodontal therapy on RA disease activity remain uncertain. RA disease activity is most often measured with the 28-joint Disease Activity Score (DAS28), which combines joint counts, the patient’s global assessment, and a blood inflammation marker, either the erythrocyte sedimentation rate (DAS28-ESR) or C-reactive protein (DAS28-CRP); the two formulations are not interchangeable, with the DAS28-CRP being systematically lower [12].

Several systematic reviews and meta-analyses have addressed the periodontal–RA question [13,14], but their methodological choices warrant reappraisal. Silva et al. [13] pooled randomized and quasi-randomized trials and combined the two DAS28 formulations within a single estimate, despite their non-interchangeability. The overview by Oliveira et al. [14] pooled primary studies of mixed design. Neither incorporates the randomized trials published since 2022, including the OPERA [15], Kaveri [16] and Nakajima [3] trials.

We therefore designed this review to (i) restrict eligibility to randomized controlled trials, (ii) analyze DAS28-CRP and DAS28-ESR as separate co-primary outcomes, and (iii) include this recent evidence. In adults with concomitant RA and periodontitis, we asked whether the first two steps of periodontal therapy (steps 1 and 2 of the 2020 EFP S3-level guideline [17]), when compared with no treatment, delayed treatment, or oral hygiene instructions alone, reduce RA disease activity.

## 2. Materials and Methods

### 2.1. Protocol and Registration

**Figure 1:**
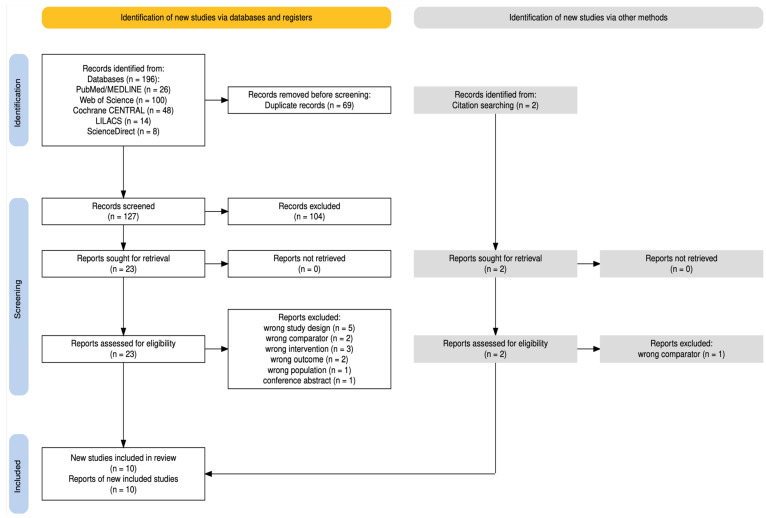
PRISMA flowchart summarizing the study identification and selection process [20].

This systematic review and meta-analysis was conducted in accordance with the methods recommended in the Cochrane Handbook for Systematic Reviews of Interventions, version 6.5 [18], and reported in accordance with the Preferred Reporting Items for Systematic Reviews and Meta-Analyses (PRISMA) 2020 statement [19]; the flow diagram (Figure 1) was produced using the PRISMA2020 Shiny web app [20] (https://estech.shinyapps.io/prisma_flowdiagram/, updated June 2022; accessed June 2026), and the completed checklist is provided in File S4. A review protocol specifying the review question, eligibility criteria, search strategy, outcomes, and planned analyses, including pre-specified subgroup and sensitivity analyses, was developed a priori and is available from the corresponding author upon reasonable request. The protocol for this review was registered in PROSPERO (registration number CRD420261400735). Deviations from the a priori protocol are declared in Section 2.9.

### 2.2. PICOS

The review question was formulated using the PICOS framework [19]: in adult patients with concomitant rheumatoid arthritis and periodontitis, does periodontal therapy reduce rheumatoid arthritis disease activity compared to no or delayed periodontal treatment?

**P—Population.** Adults with both rheumatoid arthritis and periodontitis.

**I—Intervention.** Steps 1 and 2 of the 2020 EFP S3-level clinical practice guideline [17], delivered through supragingival PMPR and subgingival instrumentation, with or without adjunctive oral hygiene instructions.

**C—Comparator.** No periodontal treatment, delayed periodontal treatment, or oral hygiene instructions alone.

**O—Outcomes.** Change from baseline in DAS28-CRP and DAS28-ESR at 3 and 6 months post-intervention, as co-primary outcomes. Secondary outcomes: probing pocket depth, clinical attachment level.

**S—Study designs.** Randomized controlled trials.

### 2.3. Eligibility Criteria

Studies were eligible for inclusion if they enrolled adults (≥18 years) with a concurrent diagnosis of rheumatoid arthritis and periodontitis. Rheumatoid arthritis had to be diagnosed according to the 1987 American Rheumatism Association criteria [21], the 2010 ACR/EULAR criteria [22], or both, applied consistently with the year of the trial. Periodontitis had to be diagnosed according to an internationally recognized classification applied consistently with the year of the trial: the 1999 Armitage classification [23], the AAP/CDC case definitions [24,25], or the 2018 World Workshop classification [26,27]. Participants had to be unmedicated or on stable pharmacological treatment for rheumatoid arthritis for at least three months prior to baseline. Only randomized controlled trials with parallel-group or delayed-start designs and with preserved randomization between immediate-treatment and delayed-treatment arms were eligible. No language or publication date restrictions were applied.

Studies were excluded if they enrolled pregnant or lactating women, edentulous participants, participants with comorbidities likely to confound the assessment of systemic inflammatory markers like other autoimmune or systemic inflammatory diseases, or participants who had received any form of professional periodontal therapy or systemic antibiotics within the three months preceding baseline. Trials with adjunctive systemic or local antimicrobials in the intervention arm were eligible and analyzed sensitively. Trials comparing two active forms of periodontal therapy without an untreated or minimally treated control arm, and trials whose control arm consisted of patients with rheumatoid arthritis without periodontitis, were excluded. Quasi-experimental designs without preserved randomization, observational study designs, case reports and series, narrative and systematic reviews, meta-analyses, editorials, in vitro and animal studies, and conference abstracts without identifiable full-text publication were also excluded.

### 2.4. Information Sources and Search Strategy

A systematic search was conducted in five electronic databases, PubMed/MEDLINE, the Cochrane Central Register of Controlled Trials (CENTRAL), Web of Science (Core Collection), ScienceDirect, and the Latin American and Caribbean Health Sciences Literature database (LILACS). Embase was not queried because the authors’ institution does not subscribe to it. The last search was run on 15 February 2026. No restrictions on language or publication date were applied. The search strategy was built around three conceptual blocks, periodontitis, rheumatoid arthritis, and periodontal therapy, which were combined with Boolean operators, and adapted to the syntax and controlled vocabulary of each database (MeSH for PubMed, DeCS for LILACS, TS= [Topic] field for Web of Science), with a NOT operator excluding animal-model and in vitro records in CENTRAL and Web of Science. The legacy terms “scaling and root planing” and “non-surgical periodontal therapy” were retained in the search blocks, as all eligible trials, including those published after the 2020 EFP S3-level guideline [17], continue to describe the delivered intervention using these terms. A supplementary PubMed search restricted to the most recent publication year and using free-text terms only was conducted to capture records not yet indexed in MEDLINE; no additional eligible records were identified and ClinicalTrials.gov was not searched directly. Because CENTRAL also incorporates records from trial registers and trial result registers [18], any trial registry records retrieved through CENTRAL corresponded either to trials already captured in the bibliographic databases or to registrations without reported results, and yielded no additional inclusions. The full search strategy for each database is provided in File S1. Electronic searches were complemented by manual screening of the reference lists of all included studies and of relevant systematic reviews. Retrieved records were exported to Zotero for deduplication and screening.

### 2.5. Selection Process

Records retrieved from all sources were exported to Zotero (Corporation for Digital Scholarship, Vienna, VA, USA), where duplicates were removed automatically and then verified manually. The remaining records were screened in two phases: titles and abstracts were first assessed against the eligibility criteria, followed by full-text retrieval and assessment of records whose titles and abstracts did not provide sufficient information for a decision or that appeared to meet the criteria. Screening was performed by one reviewer (L.K.) and verified by a second reviewer (C.R.); disagreements at either stage were resolved by discussion. Reasons for exclusion at the full-text stage were recorded and are reported in the PRISMA 2020 flow diagram (Figure 1).

### 2.6. Data Extraction

A standardized data extraction form, piloted on two studies before full deployment, was used to extract the following variables from each included trial: bibliographic information (first author, year, study design); sample size and group allocation; participant characteristics (age, sex distribution, RA diagnostic criteria, periodontitis diagnostic criteria, RA pharmacological treatment); details of the periodontal intervention and of the control condition; and outcome data (DAS28-CRP and DAS28-ESR, probing pocket depth, clinical attachment level, and bleeding on probing) at baseline and at the 3-month and 6-month timepoints. Data were extracted by one reviewer (L.K.) and verified by a second reviewer (C.R.); disagreements were resolved by discussion.

When outcome data were reported as median with interquartile range or with minimum and maximum values, mean and standard deviation were reconstructed using the methods of Luo et al. [28] and Shi et al. [29]. When data were available only in graphical form, two independent extractors (L.K. and J.B.) used WebPlotDigitizer (Ankit Rohatgi, Pacifica, CA, USA) and reached consensus on each value. Change-from-baseline standard deviations, when not reported, were imputed following Section 6.5.2.8 of the Cochrane Handbook [18]. The equations used for these reconstructions and imputations are reported in File S2.

### 2.7. Risk of Bias and Certainty of Evidence Assessment

The risk of bias in each included trial was assessed using the revised Cochrane Risk of Bias tool for randomized trials (RoB-2) [30], applied to the co-primary outcome of change from baseline in DAS28 at 3 and 6 months and targeting the effect of assignment to the interventions. Two reviewers (L.K. and C.R.) independently performed the assessments across the five RoB-2 domains; disagreements were resolved by discussion. For the trial by Pinho et al. [31], the assessment was restricted to the randomized G1 versus G2 comparison. The traffic-light plot was generated with the robvis Shiny web app [32] (https://mcguinlu.shinyapps.io/robvis/; accessed March 2026).

The potential for reporting bias within each synthesis was not assessed through funnel plots or formal statistical tests, as the number of studies contributing to each pooled estimate was below the minimum of ten recommended by the Cochrane Handbook for such assessments to be informative [18]. Trial registry records retrieved through CENTRAL included registered trials with no corresponding published results; while the reasons cannot be determined, this raises the possibility that unpublished negative findings may be absent from the present synthesis.

The certainty of the body of evidence for each co-primary outcome was rated according to the GRADE approach [33], considering within-study risk of bias, inconsistency, indirectness, imprecision, and publication bias. The corresponding summary-of-findings table is provided in Table S1.

### 2.8. Outcomes and Data Synthesis

The co-primary outcome was change from baseline in the 28-joint Disease Activity Score at 3 months post-intervention, separately analyzed for the DAS28-CRP and the DAS28-ESR. The two DAS28 formulations were treated as distinct co-primary outcomes and reported on separate forest plots; cross-formulation pooling was not performed. Change from baseline in DAS28-CRP at 6 months was pre-specified as a co-primary outcome but, for the reason given in Section 2.9, was not pooled, and is instead reported narratively.

The secondary outcomes were periodontal clinical parameters at the same timepoints: probing pocket depth (PPD, in millimeters) and clinical attachment level (CAL, in millimeters). Bleeding on probing (BOP) was pre-specified as a secondary outcome in the registered protocol but was not retained for quantitative synthesis because the units in which BOP was reported across the eligible trials were not harmonizable into a common comparative metric.

The 3- and 6-month timepoints were selected to align with the schedule of supportive periodontal care for treated periodontitis patients. Following the first two steps of therapy, the EFP S3-level guideline recommends supportive periodontal care delivered two to four times per year (i.e., at approximately 3- to 6-month intervals), tailored to the patient’s risk profile, with intervals as short as 3 months where progression control requires it [17]. As all participants in the included trials had treated or concurrent periodontitis and therefore remained at elevated risk of recurrence, these intervals define the clinically meaningful window over which a periodontal therapy effect on rheumatoid arthritis disease activity would be expected to manifest.

Quantitative synthesis was performed in Review Managers 5.4 (Cochrane, London, UK). Because the eligible trials were expected to differ in rheumatoid arthritis pharmacological regimens, periodontitis severity, periodontal treatment protocols, follow-up duration, and control conditions, a random-effects model with the DerSimonian–Laird estimator [34] was pre-specified as the primary analysis. Pooled effect estimates were calculated as mean differences (MDs) with 95% confidence intervals; the mean difference was used as the effect measure because, within each DAS28 formulation, the outcome was reported on a single common scale, so standardization was not required. Inference was based primarily on the point estimate and its 95% confidence interval; where statistical significance is referred to, a conventional two-sided threshold of *p* < 0.05 was applied. Statistical heterogeneity was quantified using the I^2^ statistic, interpreted with the Cochrane Handbook v6.5 thresholds [18], the χ^2^ test (significance threshold *p* < 0.10 given the limited number of trials per analysis), and the between-study variance Tau^2^.

When change-from-baseline standard deviations were not reported, they were derived from baseline and follow-up values using the Cochrane Handbook formula (Section 6.5.2.8) [18], assuming a pre/post correlation coefficient of r = 0.5 in the primary analysis. Robustness to this parameter was assessed in sensitivity analyses using r = 0 and r = 0.7. For de Pablo et al. [15], the follow-up DAS28-CRP standard deviation was not reported and was replaced by the baseline standard deviation, as permitted by the same section. The equations used are detailed in File S2. For Pinho et al. [31], DAS28 values were reported only for the intervention arm; the trial was therefore not estimable and contributed to neither the between-group nor the change-from-baseline analyses, remaining visible in the forest plots with zero weight.

For Thilagar et al. [35], the DAS28 formulation was not specified in the report; as C-reactive protein was the only acute-phase reactant measured and reported by the authors, the trial’s DAS28 values were assigned to the DAS28-CRP analysis.

Sensitivity analyses comprised the following: (i) exclusion of trials judged at overall high risk of bias on the RoB-2 assessment; (ii) comparison of fixed-effect and random-effects estimates; (iii) exclusion of trials in which adjunctive systemic antimicrobials were administered alongside the periodontal intervention; (iv) exclusion of trials in which the mean baseline disease activity exceeded the high-disease-activity threshold (DAS28-ESR > 5.1); and (v) recalculation of the confidence intervals of the pooled estimates using the Hartung–Knapp adjustment. As this adjustment is not implemented in Review Manager, it was computed in Microsoft Excel using the formula described in the Cochrane Handbook [18]; the full computation and per-outcome results are provided in File S3. Prediction intervals were not derived because the Cochrane Handbook recommends caution in their interpretation when fewer than approximately ten studies contribute to an estimate, a condition not met by any of the present syntheses [18].

The hypothesized dose–response relationship between periodontal improvement and rheumatoid arthritis disease activity reduction was explored at the study level by correlating the change from baseline in DAS28 with the changes in probing pocket depth and clinical attachment level. This exploratory analysis was restricted to the trials reporting these outcomes directly as mean ± standard deviation (five trials). Pearson and Spearman rank correlation coefficients were computed in IBM SPSS Statistics version 32 (IBM Corp., Armonk, NY, USA), with 95% confidence intervals derived from Fisher’s r-to-z transformation for the Pearson coefficient and the Bonett–Wright method for the Spearman coefficient; all tests were two-tailed.

### 2.9. Deviations from the a Priori Protocol

The four pre-specified subgroup analyses (pharmacological rheumatoid arthritis management, baseline periodontitis severity, type of control condition, and adjunctive antimicrobials) were not performed: classification systems and reporting categories across the eligible trials were either heterogeneous or yielded subgroups below the pre-specified threshold of three trials. The 3-month timepoint window of ±4 weeks was applied flexibly for one trial, per Ortiz et al. [8], with follow-up at 6 weeks. For Nakajima et al. [3], conventional synthetic DMARDs were initiated at baseline in participants assigned to Group A and Group B, departing from the protocol-specified criterion of stable RA treatment for at least three months prior to baseline; the trial was retained because the timing of DMARD initiation was symmetric between arms. Two sensitivity analyses not pre-specified in the protocol were conducted to explore plausible sources of heterogeneity (exclusion of trials using adjunctive systemic antimicrobials and exclusion of trials with mean baseline DAS28-ESR > 5.1). A further analysis not pre-specified in the protocol, recalculation of the pooled confidence intervals with the Hartung–Knapp adjustment, was added to assess the reliability of the confidence intervals given the small number of trials contributing to each estimate. The exclusion of bleeding on probing from quantitative synthesis is detailed in Section 2.8. The pre-specified pooling of change from baseline in DAS28-CRP at 6 months was not performed: only two trials reported this outcome (Nguyen et al. [36] and de Pablo et al. [15]), and the pooled estimate was not stable across the range of plausible pre/post correlation values (Section 3.4.3); the two trials are therefore reported narratively at this timepoint rather than combined in a meta-analysis. Change from baseline in DAS28-ESR at 6 months was also not pooled due to lack of data available at 6 months.

## 3. Results

### 3.1. Study Selection

A total of 196 records were identified across five electronic databases using the search strategies and filters detailed in File S1. After removal of 69 inter-database duplicates, 127 records were screened on title and abstract, of which 104 were excluded. Twenty-three reports were sought and retrieved for full-text assessment, supplemented by two reports identified through citation searching of the reference lists of included trials and of relevant systematic reviews and meta-analyses. The 15 reports excluded at full-text assessment, with reasons, are listed in Table S2. Ten randomised controlled trials met the inclusion criteria and were included in the systematic review and meta-analysis, as illustrated in Figure 1.

### 3.2. Characteristics of the Included Studies

The main characteristics of the 10 included trials are summarised in Table 1.

### 3.3. Risk of Bias and Certainty of Evidence 

**Figure 2:**
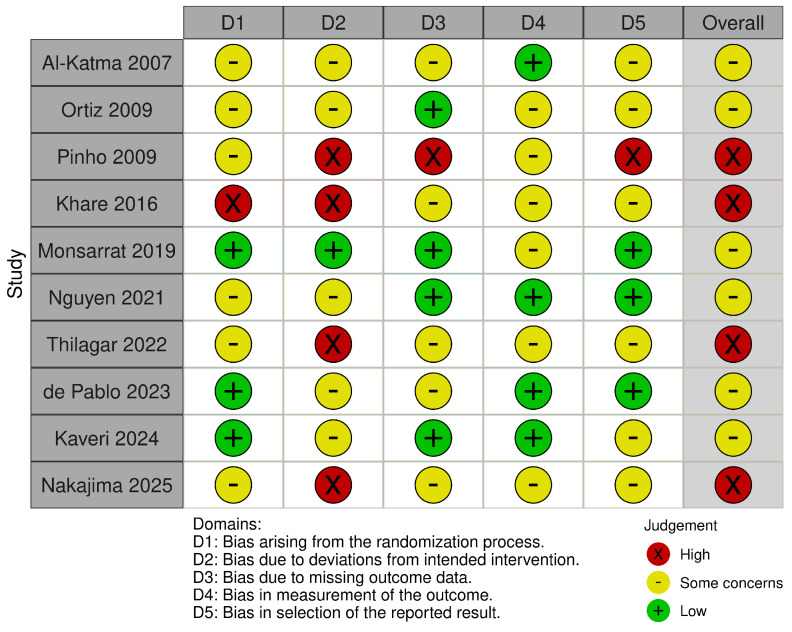
Risk of bias in the included trials, assessed with the revised Cochrane risk-of-bias tool [3,8,15,16,31,35,36,37,38,39].

Risk of bias was assessed with the revised Cochrane RoB-2 tool [30], applied to the co-primary outcome of change from baseline in DAS28 at 3 and 6 months and targeting the effect of assignment to the interventions. For Pinho et al. [31], the assessment was restricted to the randomized G1 versus G2 comparison. None of the ten trials was judged at overall low risk of bias. Six trials [8,15,16,36,37,39] received an overall judgement of some concerns, mostly driven by incomplete reporting of the randomization procedure (D1) and open-label delivery of the periodontal intervention (D2). Four trials [3,31,35,38] received an overall judgement of high risk of bias. The per-trial domain-level judgements are displayed in the RoB-2 traffic light plot (Figure 2), generated with the robvis Shiny web app [32]. Rated with GRADE, the certainty of evidence was low for the DAS28-CRP estimates and very low for the DAS28-ESR estimates at 3 months, downgraded for risk of bias, imprecision and, for DAS28-ESR, substantial inconsistency; the summary-of-findings table is provided in Table S1.

### 3.4. DAS28 Meta-Analyses

The 28-joint Disease Activity Score (DAS28) was analysed separately for its two formulations (DAS28-ESR and DAS28-CRP). DAS28-ESR was analysed at 3 months only; no included trial reported DAS28-ESR at 6 months. DAS28-CRP was analysed at 3 months; the 6-month timepoint is reported narratively. For each comparison, two mean differences (MD) are reported. The first compares the intervention and control groups on their final scores at follow-up. The second compares the change each group underwent from its own baseline. The two can differ when the groups were not balanced at the start: the follow-up comparison is then distorted by those initial differences, whereas the change-from-baseline comparison reduces the influence of baseline imbalance by referencing each group to its own starting value. Consistent with the registered co-primary outcome, the change-from-baseline estimate was taken as the primary basis for interpretation, while remaining sensitive to the assumed pre/post correlation used to impute missing change-score standard deviations (Section 2.8). Negative values favour the periodontal intervention. To interpret these magnitudes clinically, we used the EULAR response criteria, the standard rheumatological reference for grading change in disease activity, which apply to both DAS28 formulations: a reduction exceeding 0.6 units marks a perceptible improvement and one exceeding 1.2 units a substantial improvement [40]. These thresholds are conventionally applied at the individual patient level; here they contextualise group-level mean differences and do not indicate the proportion of patients achieving a clinically meaningful response.

#### 3.4.1. DAS28-ESR at 3 Months

**Figure 3:**
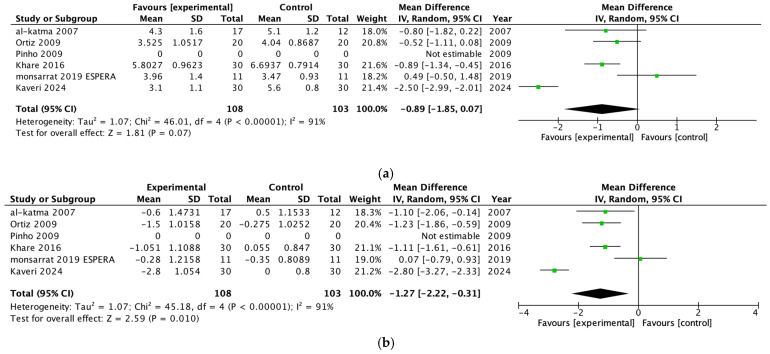
Forest plot of periodontal therapy versus standard rheumatoid arthritis care, outcome DAS28-ESR at 3 months [8,16,31,37,38,39]. If there are multiple panels, they are listed as (**a**) between-group difference at follow-up and (**b**) between-group difference in change from baseline. Negative values favour periodontal therapy.

Six trials [8,16,31,37,38,39] were eligible for the DAS28-ESR analysis at 3 months. The trial of Pinho et al. [31] was not estimable (Section 2.8), leaving five trials contributing quantitatively (108 intervention vs 103 control participants for the between-group analysis at follow-up; 108 vs 103 for the change-from-baseline analysis). The between-group difference at follow-up was −0.89 (95% CI −1.85 to 0.07; *p* = 0.07) (Figure 3a) and the between-group difference in change from baseline was −1.27 (95% CI −2.22 to −0.31; *p* = 0.010) (Figure 3b). Both estimates favoured periodontal therapy; the change-from-baseline estimate exceeded the EULAR threshold for substantial clinical improvement, while the between-group estimate at follow-up was of comparable magnitude but did not reach the conventional significance threshold. Between-study heterogeneity was high in both analyses (I^2^ = 91%).

#### 3.4.2. DAS28-CRP at 3 Months

**Figure 4:**
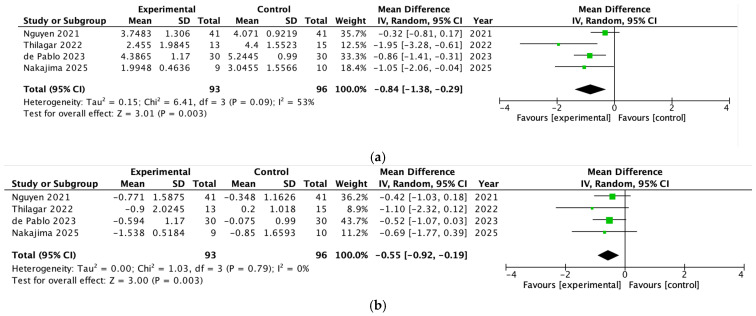
Forest plot of periodontal therapy versus standard rheumatoid arthritis care, outcome DAS28-CRP at 3 months [3,15,35,36]. If there are multiple panels, they are listed as (**a**) between-group difference at follow-up and (**b**) between-group difference in change from baseline. Negative values favour periodontal therapy.

Four trials [3,15,35,36] contributed to the DAS28-CRP analysis at 3 months (93 intervention vs 96 control participants). The between-group difference at follow-up was −0.84 (95% CI −1.38 to −0.29; *p* = 0.003; I^2^ = 53%) (Figure 4a), and the between-group difference in change from baseline was −0.55 (95% CI −0.92 to −0.19; *p* = 0.003; I^2^ = 0%) (Figure 4b). Both estimates were on the favourable side of the EULAR threshold for perceptible clinical improvement.

#### 3.4.3. DAS28-CRP at 6 Months

Evidence at 6 months was sparse: only two of the included trials extended follow-up to this timepoint (Nguyen et al. [36] and de Pablo et al. [15]), the remaining trials reporting outcomes at 3 months or earlier. This scarcity is consequential because the temporal pattern of any effect on rheumatoid arthritis disease activity may not be fully captured at 3 months. In Nguyen et al. [36], the reduction in DAS28-CRP within the intervention arm was already significant at 3 months relative to baseline; however, the between-group difference in DAS28-CRP reached statistical significance only at 6 months (*p* = 0.013). The question of whether the between-group separation observed by Nguyen et al. [36] at 6 months reflects a genuinely delayed or cumulative effect, or the limitations of a two-trial comparison, cannot be resolved from the present data. Adequately powered trials with follow-up beyond 3 months are needed to characterise the time course of the response. When the two trials were nonetheless combined for inspection, the pooled change-from-baseline estimate was significant under the primary correlation assumption (r = 0.5; MD −0.57, 95% CI −1.01 to −0.14) and under r = 0.7 (MD −0.57, 95% CI −0.91 to −0.23), but crossed the null under the maximally conservative assumption r = 0 (MD −0.58, 95% CI −1.18 to +0.03). Because this conclusion rested on only two trials and was not stable across the full range of plausible correlation values, the two trials are reported narratively at this timepoint rather than pooled.

### 3.5. Sensitivity Analyses

Sensitivity analyses (fixed-effect model; exclusion of high-risk-of-bias trials, of trials with adjunctive antimicrobials, of trials with baseline DAS28-ESR above 5.1, and of Nakajima et al. [3] from the DAS28-CRP analyses; variation of the pre/post correlation coefficient) did not alter the direction of the 3-month estimates, and the DAS28-CRP estimates remained significant throughout.

Specifically, excluding Nakajima et al. [3] left the DAS28-CRP estimates essentially unchanged at 3 months (follow-up: MD −0.83, 95% CI −1.50 to −0.15, *p* = 0.016, I^2^ = 65%; change-from-baseline: MD −0.51, 95% CI −0.90 to −0.13, *p* = 0.010, I^2^ = 0%). Excluding the four trials judged at high risk of bias also preserved the direction and significance of the DAS28-CRP estimates (follow-up: MD −0.58, 95% CI −1.09 to −0.07, *p* = 0.026, I^2^ = 48%; change-from-baseline: MD −0.48, 95% CI −0.88 to −0.07, *p* = 0.022, I^2^ = 0%), indicating that the DAS28-CRP signal does not depend on the inclusion of Nakajima et al. [3] nor on the high-risk-of-bias trials (including Thilagar et al. [35], whose values were reconstructed from median–range data).

For the DAS28-ESR outcome (primary analysis I^2^ = 91%), excluding trials with adjunctive antimicrobials (MD −1.14, 95% CI −1.51 to −0.78, I^2^ = 0%) or with baseline DAS28-ESR above 5.1 reduced heterogeneity (to I^2^ = 0% and 67%, respectively) without reversing the direction, though on fewer trials.

Under the Hartung–Knapp adjustment, the confidence intervals of all pooled estimates widened, and only the DAS28-CRP change-from-baseline estimate at 3 months retained statistical significance (MD −0.55, 95% CI −0.90 to −0.21; *p* = 0.014). The other 3-month estimates no longer reached significance (DAS28-CRP follow-up −0.84, 95% CI −1.77 to 0.09; DAS28-ESR change −1.27, 95% CI −2.54 to 0.01), and the 6-month estimates, based on two trials each, could not be evaluated by this method.

### 3.6. Secondary Outcomes: Periodontal Clinical Parameters and Exploratory Dose–Response Analysis

In every trial reporting probing pocket depth (PPD) and clinical attachment level (CAL) at 3 months, both decreased in the intervention arm (PPD reductions from −0.21 mm Ortiz [8] to −0.94 mm Khare [38]; CAL from −0.13 mm to −0.75 mm), confirming that the delivered therapy improved periodontal status. We then explored, at the study level, whether the degree of periodontal improvement tracked the reduction in DAS28 (Pearson and Spearman correlations; Table 2). No correlation between ΔDAS28 and either ΔPPD or ΔCAL reached statistical significance; only five trials contributed, limiting the power of this exploratory analysis.

## 4. Discussion

This systematic review and meta-analysis of ten randomized controlled trials evaluated whether the first two steps of periodontal therapy modify rheumatoid arthritis (RA) disease activity in patients with concomitant periodontitis. The pooled estimates pointed in a consistent direction across both formulations of the DAS28 and both analytical approaches. The effect reached statistical significance on DAS28-CRP at three months and on the change-from-baseline DAS28-ESR; the DAS28-ESR follow-up estimate was concordant in direction but did not reach significance. The convergence of direction across the four pooled analyses is already informative. DAS28-ESR and DAS28-CRP are not interchangeable scores and behave differently in calibrated populations [12]; finding a comparable directional effect on both, and across two distinct analytical formulations, makes it unlikely that the signal is a metric-specific artefact or an artefact of baseline imbalance. PPD and CAL decreased in every intervention arm, so the partial inferential weakness on the ESR between-group estimate cannot be attributed to ineffective periodontal therapy.

The two analytical approaches nonetheless diverged on the ESR metric: the change-from-baseline analysis was significant whereas the follow-up analysis was not. As noted in Section 3.4, this pattern points to baseline DAS28-ESR imbalance between arms in some trials, which the change-from-baseline analysis removes. Given the high heterogeneity (I^2^ = 91%) and few trials, this divergence cannot be fully resolved here and the ESR estimates warrant caution.

These findings should be read alongside the existing evidence, with which they are broadly compatible but not redundant. As noted above, Silva et al. [13], pooled trials of mixed design and combined the DAS28 formulations, whereas the umbrella review by Oliveira et al. [14] reported within-arm pre/post estimates rather than between-group contrasts. Each synthesis answers a slightly different question, and the present analysis, restricted to randomized evidence, with the two formulations kept separate and incorporating the recent OPERA [15], Kaveri [16], and Nakajima [3] trials, provides a more conservative inferential framework while reaching directionally similar conclusions.

The contrast with the ESPERA trial [39] is instructive. ESPERA was stopped for futility after 22 participants and reported a between-group DAS28-ESR difference of essentially zero, a result often read as evidence that periodontal therapy does not affect RA disease activity. Seen alongside the present meta-analysis, it rather illustrates how a small trial can return a null estimate in the presence of a real but modest effect, especially in a population whose inflammatory burden was already controlled pharmacologically. The pooled evidence does not overturn ESPERA but makes its null result interpretable rather than definitive.

A second axis along which these results deserve interpretation is the dose–response question. At the study level, no correlation was detectable between change in DAS28 and change in either PPD or CAL. The temptation is to read this as an absence of a biological link, but several elements caution against that reading. Study-level correlations across a handful of heterogeneous trials are notoriously underpowered to detect anything but very strong effects, and the wider observational literature consistently shows that periodontal severity tracks RA disease activity at the individual level. Hussain et al. [7] have reported that periodontitis was found to be associated with a higher DAS28 in case–control studies; Rodríguez-Lozano et al. [6] and Karapetsa et al. [41], found adjusted odds ratios of comparable magnitude for active RA in patients with periodontitis; and Silva et al. [42] noted that the largest DAS28-CRP reductions occurred in patients with the most severe baseline periodontal involvement. The more plausible reading is that the dose–response relationship remains unresolved at the study level and that its characterization will require patient-level data, rather than that the observational signal seen at the individual level is biologically absent.

The clinical implications of these findings should be framed carefully. RA is not curable, and any non-pharmacological intervention is unlikely to substitute for disease-modifying treatment; the question is whether it earns a place alongside it. The magnitudes observed here meet or approach the EULAR threshold for perceptible improvement, and the change-from-baseline DAS28-ESR estimate exceeds the threshold for substantial improvement. In practice, this can mark the difference between moderate and low disease activity.

This clinical magnitude should not, however, eclipse the patient-centered dimension, which was rarely assessed. Among the included trials, only Monsarrat et al. [39] formally measured quality of life, finding no significant effect on the HAQ or the overall GOHAI (a non-significant positive trend was confined to the psychosocial domain; adjusted mean difference (aMD) 0.13, 95% CI −0.07 to 0.33). This null result is difficult to interpret in isolation, as ESPERA also showed the smallest reduction in DAS28-ESR of any included trial (aMD −0.03, 95% CI −0.98 to 0.92), consistent with a low margin for inflammatory improvement in its largely biologic-DMARD-treated population. Quality of life in patients in whom periodontal therapy does lower disease activity therefore remains untested and would be best addressed by a trial enrolling such responders.

Several limitations temper the certainty of these conclusions. Heterogeneity was substantial for the DAS28-ESR analyses at three months, reflecting genuine variability in study populations, periodontal protocols (with or without adjunctive antimicrobials), and concurrent pharmacological RA regimens that no statistical pooling can fully reconcile. The number of trials contributing to each estimate was small, particularly at six months, and four of the ten included trials carried a high overall risk of bias on RoB-2, with six raising some concerns; the recurrent issues were the randomization procedure and the open-label nature of the intervention. Embase could not be queried because institutional access was unavailable, and the values for Thilagar et al. [35] were reconstructed from median–range data using the Luo–Shi [28,29] estimators. Pinho et al. [31], not estimable for lack of control-arm data, contributed no weight to any pooled estimate. None of these limitations is unique to the field, but their cumulative effect is to make the present synthesis a careful confirmation of a directionally consistent effect rather than a definitive demonstration of its magnitude.

Notably, excluding trials at high risk of bias did not reduce heterogeneity in the present analysis (I^2^ = 93%), in contrast to Silva et al. [13], who attributed sizeable reductions in heterogeneity to selection and reporting bias; in our data, study quality does not appear to be the principal driver of between-study variability.

A further limitation concerns the level at which the EULAR improvement threshold can be interpreted. The present synthesis applies that threshold to pooled group-level mean differences, which indicate the average shift in disease activity but not the proportion of individual patients achieving a clinically meaningful response. The included trials did not report responder classifications, so the number of patients crossing the threshold for genuine clinical improvement, as distinct from a statistically detectable group-level change, cannot be derived. Trials reporting individual-level response categories would allow this distinction to be addressed in future work.

Future trials should aim for greater standardization of the periodontal intervention according to the 2020 EFP S3-level clinical practice guideline [17], document stability of the RA pharmacological regimen throughout follow-up, extend the observation window to at least six months, and report outcomes at the individual level to permit a proper assessment of the dose–response relationship, including stratification by baseline periodontal severity.

Two structural obstacles will continue to constrain trial design. First, blinding is intrinsically limited: a hands-on periodontal intervention cannot be concealed from the operator and is difficult to mask from the participant, so blinding of the outcome assessor is the realistic ceiling. Second, withholding periodontal care for six months or more to maintain a true no-treatment control raises ethical concerns, which most recent trials address by providing oral hygiene instructions or a delayed-treatment design. These constraints are inherent to the question, not deficiencies in trial conduct.

## 5. Conclusions

In adults with rheumatoid arthritis and concomitant periodontitis, the first two steps of periodontal therapy may reduce DAS28-CRP in the short term, compared with no periodontal treatment, delayed treatment, or oral hygiene instructions alone. The signal was directionally consistent across both DAS28 formulations and both analytical approaches but reached robust significance only for the change-from-baseline DAS28-CRP at 3 months under a conservative (Hartung–Knapp) analysis; the DAS28-ESR estimates were limited by substantial heterogeneity, and any effect beyond 3 months remains unresolved (two trials). Because the analysis pooled group-level mean differences without individual responder data, it cannot quantify the proportion of patients achieving a clinically meaningful improvement. Establishing whether periodontal therapy yields a predictable adjunctive benefit on RA disease activity will require adequately powered trials reporting patient-level outcomes.

## Figures and Tables

**Table 1:** Characteristics of the included trials.

Study (Year)	Design	*n* (Int/Ctrl)	Female % (Int/Ctrl)	Mean Age, y (Int/Ctrl)	RA Criteria	Periodontitis Criteria	Intervention	Control	Follow-Up (Months)
Al-Katma 2007 [37]	Pilot RCT, assessor-blinded	17/12	88/83	55.0 ± 9.4/51.7 ± 12.3	ARA 1987 [21] (DAS28 ≥ 2.5)	Armitage 1999 [23]; mild–moderate, ≥3years, ≥20 teeth	PMPR + subgingival instrumentation + OHI	No periodontal treatment, no OHI	2
Ortiz 2009 [8]	Four-arm RCT	20/20	80/95	55.5 (39–87) median (range)	(clinical)	Severe chronic periodontitis (clinical); ≥20 teeth	PMPR + subgingival instrumentation + OHI	Delayed periodontal treatment	1.5
Pinho 2009 [31]	RCT G1 vs G2 (nested in 5-group study)	15/15	60 overall	50 (range 35–60) overall	ARA 1987 [21]	Machtei 1992 (CAL ≥ 6 mm + PPD ≥ 5 mm)	PMPR + subgingival instrumentation	No periodontal treatment	6
Khare 2016 [38]	Two-group parallel comparison	30/30	83/87	49 ± 10.3/51 ± 9.2	ARA 1987 [21]	Generalized periodontitis Carranza’s clinical periodontology 2006	PMPR + subgingival instrumentation + OHI	No periodontal treatment	3
Monsarrat 2019 (ESPERA) [39]	Open-label delayed-start RCT	11/11	55/73	64.7 ± 5.7/58.5 ± 8.7	ARA 1987 [21] (DAS28-ESR 3.2–5.1)	Armitage 2004 (CAL ≥ 3 mm + PPD ≥ 4 mm)	Full-mouth disinfection + PMPR + subgingival instrumentation + amoxicillin or clindamycin + CHX irrigation	Delayed periodontal treatment	3
Nguyen 2021 [36]	RCT, sealed-envelope allocation	41/41	88/93	52.9 ± 8.2/51.9 ± 9.0	ACR/EULAR 2010 [22]	Machtei (>4 teeth)	PMPR + subgingival instrumentation	OHI only	3 and 6
Thilagar 2022 [35]	Double-blind RCT	13/15	69/93	38.1 ± 9.2/46.7 ± 13.5	ARA 1987+ ACR/EULAR 2010 [21,22]	Armitage 1999 (PPD ≥ 5 mm + CAL ≥ 4 mm) [23];	PMPR + subgingival instrumentation + OHI	No periodontal treatment	2–3
de Pablo 2023 (OPERA) [15]	Feasibility delayed-start RCT	30/30	67/83	59 / 57	ARA 1987+ Aletaha 2010 [21,22]	Papapanou 2018 and Dietrich 2008 (Stage II–IV; CAL ≥ 4 mm + cumulative PPD ≥ 40 mm) [27]	Subgingival PMPR (ultrasonic + hand) + OHI coaching	OHI at baseline; delayed periodontal therapy at 6 months (study completion); 3-month CAL-based rescue	3 and 6
Kaveri 2024 (RACP) [16]	Delayed-start RCT, single-blind assessor	30/30	≈83 overall	30–65 range overall	ACR/EULAR 2010 [22]	Clinical (PPD > 3 mm + CAL > 2 mm); ≥8 teeth	PMPR + subgingival instrumentation + CHX + amoxicillin + metronidazole (5 d)	Delayed periodontal treatment	3
Nakajima 2025 [3]	Three-arm hybrid study; randomized A vs B only	9/10	89/60	68 (55–71)/71 (67–74) median (IQR)	ARA 1987 [21] (≥4 of 7 criteria)	CDC/AAP case definition [24]	PMPR + subgingival instrumentation + OHI	RA treatment alone for 3 months, then PMPR + subgingival instrumentation	3, 6, 9, 12

Abbreviations: AAP, American Academy of Periodontology; ACR, American College of Rheumatology; ARA, American Rheumatism Association; CDC, Centers for Disease Control and Prevention; CHX, chlorhexidine; EULAR, European Alliance of Associations for Rheumatology; OHI, oral hygiene instruction; PMPR, professional mechanical plaque removal. Other abbreviations are defined in the main text.

**Table 2:** Exploratory study-level correlations between the changes in DAS28, PPD, and CAL at 3 months (Pearson and Spearman, *N* = 5).

Variable Pair	Method	Coefficient	95% CI	*p* Value
ΔDAS28 vs ΔPPD	Pearson	0.127	−0.850 to 0.908	0.838
	Spearman	0.200	−0.833 to 0.922	0.747
ΔDAS28 vs ΔCAL	Pearson	0.033	−0.875 to 0.889	0.958
	Spearman	0.100	−0.859 to 0.903	0.873
ΔPPD vs ΔCAL	Pearson	0.956	0.470 to 0.997	0.011
	Spearman	0.900	−0.169 to 0.996	0.037

ΔDAS28, change from baseline in the 28-joint Disease Activity Score; ΔPPD, change in probing pocket depth; ΔCAL, change in clinical attachment level. Confidence intervals derived using Fisher’s r-to-z transformation (Pearson) and the Bonett–Wright method (Spearman); two-tailed.

## Data Availability

No new data were created.

## Supplementary Materials

The following supporting information can be downloaded at https://www.mdpi.com/article/10.3390/jcm15135099/s1. File S1: Search strategy for all databases; File S2: Equations used to reconstruct the mean and standard deviation from the median and interquartile range; File S3: Sensitivity analysis—Hartung–Knapp adjustment; File S4: PRISMA 2020 checklist; Table S1: Summary of findings (GRADE certainty of evidence); Table S2: Studies excluded at full-text assessment, with reasons for exclusion.

## Abbreviations

The following abbreviations are used in this manuscript:

AAP	American Academy of Periodontology
ACPA	Anti-citrullinated protein antibody
ACR	American College of Rheumatology
ARA	American Rheumatism Association
BOP	Bleeding on probing
CAL	Clinical attachment level
CDC	Centers for Disease Control and Prevention
CHX	Chlorhexidine
CI	Confidence interval
CRP	C-reactive protein
DAS28	Disease Activity Score on 28 joints
DMARD	Disease-modifying antirheumatic drug
EFP	European Federation of Periodontology
ESR	Erythrocyte sedimentation rate
EULAR	European Alliance of Associations for Rheumatology
GOHAI	General Oral Health Assessment Index
GRADE	Grading of Recommendations Assessment, Development and Evaluation
HAQ	Health Assessment Questionnaire
IL	Interleukin
MD	Mean difference
OHI	Oral hygiene instruction
OPG	Osteoprotegerin
PICOS	Population, Intervention, Comparator, Outcome, Study design
PMPR	Professional mechanical plaque removal
PPAD	Peptidylarginine deiminase
PPD	Probing pocket depth
PRISMA	Preferred Reporting Items for Systematic Reviews and Meta-Analyses
RA	Rheumatoid arthritis
RANKL	Receptor activator of nuclear factor-κB ligand
RCT	Randomized controlled trial
RoB-2	Revised Cochrane risk-of-bias tool for randomized trials
TNF	Tumor necrosis factor
