# Supplementary material for: The Impact of Periodontal Therapy on Disease Activity in Patients with Rheumatoid Arthritis and Concomitant Periodontitis: A Systematic Review and Meta-Analysis
Source: J Clin Med. 2026 Jun 30;15(13):5099. doi: 10.3390/jcm15135099 (PMC13362638; doi:10.3390/jcm15135099)
Supplement: Supplementary file 1 [file jcm-15-05099-s001.zip › File_S1_Search_strategy.pdf]

File S1. Search strategy for all databases

Effect of periodontal therapy in patients with rheumatoid arthritis

Population (P) Patients with rheumatoid arthritis and periodontitis
Intervention (I) Steps 1 and 2 of the 2020 EFP S3-level clinical practice guideline, delivered through supragingival PMPR and subgingival instrumentation, with or without adjunctive oral hygiene instructions
Comparator (C) No periodontal treatment / delayed treatment / oral hygiene only
Outcomes (O) Rheumatologic and secondarily periodontal clinical parameters (DAS28, PPD, CAL)

Study design : Randomized controlled trials
Databases : LILACS, PubMed, Cochrane Library, Web of Science, Science Direct

The search strategies below were adapted to the syntax and controlled vocabulary of each database to combine the three conceptual blocks: periodontitis AND rheumatoid arthritis AND periodontal therapy. No restrictions on language or publication date were applied.
In addition to the search strings above, a single native publication-/document-type filter was applied in each database, as follows:

- **PubMed/MEDLINE** — Article type: *Clinical Trial*
- **Cochrane CENTRAL** — Record type: *Trials*
- **LILACS** — Publication type: *Controlled clinical trial*
- **Web of Science** — Document type: *Article*
- **ScienceDirect** — Article type: *Research articles*

PubMed

Combines MeSH controlled vocabulary with Title/Abstract free-text terms to maximise sensitivity.

((periodontitis[MeSH Terms] OR chronic periodontitis[MeSH Terms] OR aggressive periodontitis[MeSH Terms] OR periodontal attachment loss[MeSH Terms] OR periodontal diseases[MeSH Terms] OR periodontal pocket[MeSH Terms] OR alveolar bone loss[MeSH Terms] OR tooth loss[MeSH Terms] OR periodontitis[Title/Abstract] OR periodontal[Title/Abstract])

AND (arthritis, rheumatoid[MeSH Terms] OR "rheumatoid arthritis"[Title/Abstract] OR rheumatoid[Title/Abstract] OR RA[Title/Abstract])

AND (periodontal debridement[MeSH Terms] OR dental scaling[MeSH Terms] OR root planing[MeSH Terms] OR dental prophylaxis[MeSH Terms] OR oral hygiene[MeSH Terms] OR "scaling and root planing"[Title/Abstract] OR SRP[Title/Abstract] OR "periodontal treatment"[Title/Abstract] OR "periodontal therapy"[Title/Abstract] OR "subgingival debridement"[Title/Abstract]))

Web of Science

Uses the TS= (Topic) field, which searches title, abstract, and author keywords. The term

"chronic periodontitis" was omitted from this database, as empirical testing confirmed that its inclusion did not retrieve any additional records beyond those already captured by "periodontitis" and related synonyms without sensitivity loss. Animal/in-vitro studies are excluded by the NOT operator above. A document-type filter (*Article*) was applied through the Web of Science refinement panel; study design (RCT) was assessed at the screening stage.

TS=(periodontitis OR "aggressive periodontitis" OR "periodontal attachment loss" OR "periodontal diseases" OR "periodontal pocket" OR "alveolar bone loss" OR "tooth loss")

AND TS=("rheumatoid arthritis" OR rheumatoid OR RA)

AND TS=("periodontal treatment" OR "periodontal therapy" OR "periodontal debridement" OR "scaling and root planing" OR SRP OR "dental scaling" OR "root planing")

AND TS=(human OR humans OR patient OR patients OR adult OR adults)

NOT TS=(mouse OR mice OR murine OR rat OR rats OR rodent OR animal OR animals

OR "in vitro")

Science Direct

Science Direct limits Boolean searches to a maximum of 8 logical operators per query; the strategy is therefore simplified while preserving the three conceptual blocks.

("periodontitis" OR "periodontal disease") AND ("rheumatoid arthritis") AND ("periodontal treatment" OR "scaling and root planing" OR "periodontal therapy")

LILACS

Latin American and Caribbean Health Sciences Literature. Uses DeCS/MeSH descriptors (MH:) and free text words (TW:) in Portuguese, Spanish and English.

(MH:"Periodontite" OR MH:"Doenças Periodontais" OR MH:"Periodontitis" OR MH:"Periodontal Diseases" OR MH:"Periodontitis Crônica" OR MH:"Perda da Inserção Periodontal" OR MH:"Bolsa Periodontal" OR MH:"Perda do Osso Alveolar" OR MH:"Perda de Dente" OR TW:periodontitis OR TW:periodontite OR TW:periodontal OR TW:"doença periodontal" OR TW:"enfermedad periodontal")

AND (MH:"Artrite Reumatoide" OR MH:"Arthritis, Rheumatoid" OR MH:"Artritis Reumatoide" OR TW:"rheumatoid arthritis" OR TW:"artrite reumatoide" OR TW:"artritis reumatoide" OR TW:rheumatoid)

AND (MH:"Desbridamento Periodontal" OR MH:"Raspagem Dentária" OR MH:"Aplainamento Radicular" OR MH:"Higiene Bucal" OR MH:"Periodontal Debridement" OR MH:"Dental Scaling" OR MH:"Root Planing" OR MH:"Oral Hygiene" OR TW:"tratamento periodontal" OR TW:"terapia periodontal" OR TW:"raspagem e alisamento radicular" OR TW:"alisamento radicular" OR TW:"periodontal treatment" OR TW:"periodontal therapy" OR TW:"scaling and root planing" OR TW:"tratamiento periodontal" OR TW:"terapia periodontal")

Cochrane Library (CENTRAL)

Search of the Cochrane Central Register of Controlled Trials, focused on randomized evidence. A NOT operator excludes animal-model and in vitro controlled trials, which are indexed alongside human RCTs in CENTRAL.

("rheumatoid arthritis" OR rheumatoid OR RA OR DAS28)

AND ("periodontitis" OR "periodontal disease" OR "periodontal diseases" OR "porphyromonas gingivalis" OR "attachment loss")

AND ("periodontal treatment" OR "periodontal therapy" OR "periodontal debridement" OR "scaling and root planing" OR SRP OR "dental scaling" OR "root planing")

NOT ("murine" OR "mouse" OR animal* OR "in vitro" OR rat* OR "mice")
