# Supplementary material for: The Impact of Periodontal Therapy on Disease Activity in Patients with Rheumatoid Arthritis and Concomitant Periodontitis: A Systematic Review and Meta-Analysis
Source: J Clin Med. 2026 Jun 30;15(13):5099. doi: 10.3390/jcm15135099 (PMC13362638; doi:10.3390/jcm15135099)
Supplement: Supplementary file 1 [file jcm-15-05099-s001.zip › File_S2_Equations.pdf]

File S2. Equations used to reconstruct the mean and standard deviation from the median and interquartile range

Equations used to reconstruct the mean and standard deviation from the median and interquartile range

(A) Estimation of the mean from the median and interquartile range (Luo et al., 2018)

$$\bar{X} \approx w \cdot \frac{q_1 + q_3}{2} + (1 - w) \cdot m$$

$$w = 0.7 + \frac{0.39}{n}$$

where $\bar{X}$ = estimated mean; q_1 = first quartile; m = median; q_3 = third quartile; n = sample size; w = weight assigned to the mid-quartile range, depending on the sample size.

(B) Estimation of the standard deviation from the interquartile range (Shi et al., 2020)

$$S \approx \frac{q_3 - q_1}{2\,\Phi^{-1}(\frac{0.75n - 0.125}{n + 0.25})}$$

where S = estimated standard deviation; q_1 = first quartile; q_3 = third quartile; $q_3 - q_1$ = interquartile range; n = sample size; Φ^{-1} = inverse cumulative distribution function of the standard normal distribution.

References

1. Luo, D.; Wan, X.; Liu, J.; Tong, T. Optimally Estimating the Sample Mean from the Sample Size, Median, Mid-Range, and/or Mid-Quartile Range. *Stat. Methods Med. Res.* **2018**, *27*, 1785–1805. https://doi.org/10.1177/0962280216669183.
2. Shi, J.; Luo, D.; Weng, H.; Zeng, X.-T.; Lin, L.; Chu, H.; Tong, T. Optimally Estimating the Sample Standard Deviation from the Five-Number Summary. *Res. Synth. Methods* **2020**, *11*, 641–654. https://doi.org/10.1002/jrsm.1429.
