# Supplementary material for: The Impact of Periodontal Therapy on Disease Activity in Patients with Rheumatoid Arthritis and Concomitant Periodontitis: A Systematic Review and Meta-Analysis
Source: J Clin Med. 2026 Jun 30;15(13):5099. doi: 10.3390/jcm15135099 (PMC13362638; doi:10.3390/jcm15135099)
Supplement: Supplementary file 1 [file jcm-15-05099-s001.zip › File_S3_HKSJ_sensitivity.pdf]

## File S3. Sensitivity analysis: Hartung–Knapp adjustment

*Supplementary justification prepared in response to Reviewer 1, major concern #5 (reliability of confidence intervals from the DerSimonian–Laird model with few studies per analysis).*

**Provenance and reproduction check.** Per-study, per-arm data (mean, standard deviation, sample size) were extracted from the registered RevMan 5.4 analysis file. Re-fitting the DerSimonian–Laird (DL) random-effects model from these inputs reproduced every published pooled estimate exactly (the "DL 95% CI (P)" column in Table 1 corresponds to the values reported in the manuscript), confirming the inputs prior to any conservative adjustment. The main analysis is retained as DerSimonian–Laird; the Hartung–Knapp–Sidik–Jonkman (HKSJ) adjustment was computed in Microsoft Excel using the formula of the Cochrane Handbook [18] and is reported here as a sensitivity analysis only, because RevMan 5.4 does not implement it.

**Table 1.** Pooled estimates: DerSimonian–Laird vs Hartung–Knapp.

| Outcome (3 and 6 months) | k | $I^2$ | $\tau^2$ | MD | DL 95% CI (P) | HKSJ 95% CI (P) |
|---|---|---|---|---|---|---|
| DAS28-ESR, 3 m, follow-up | 5 | 91% | 1.066 | −0.89 | [−1.85; 0.07] (0.07) | [−2.23; 0.45] (0.14) |
| DAS28-CRP, 3 m, follow-up | 4 | 53% | 0.154 | −0.84 | [−1.38; −0.29] (0.003) | [−1.77; 0.09] (0.06) |
| DAS28-ESR, 3 m, change | 5 | 91% | 1.069 | −1.27 | [−2.22; −0.31] (0.010) | [−2.54; 0.01] (0.051) |
| **DAS28-CRP, 3 m, change** | **4** | **0%** | **0** | **−0.55** | **[−0.92; −0.19] (0.003)** | **[−0.90; −0.21] (0.014)** |

*MD, mean difference (intervention − comparator), in DAS28 units; negative values favour periodontal therapy. k, number of pooled studies. The row in bold (DAS28-CRP, 3 m, change-from-baseline) is the only estimate that remains statistically significant under the conservative HKSJ adjustment.*

### Methods and formulas

**Per study:** $MD_i$ = mean_int − mean_comp; $v_i = SD\_int^2/n\_int + SD\_comp^2/n\_comp$.

**DerSimonian–Laird:** between-study variance $\tau^2$ by the method-of-moments estimator; random-effects weights $w_i^* = 1/(v_i + \tau^2)$; summary $\mu = \Sigma\, w_i^* \cdot MD_i / \Sigma\, w_i^*$; 95% CI = $\mu \pm 1.96 \cdot SE(\mu)$.

**Hartung–Knapp–Sidik–Jonkman:** same point estimate $\mu$; $SE\_HK = \sqrt{[\, \Sigma\, w_i^* \cdot (MD_i - \mu)^2 / ((k-1) \cdot \Sigma\, w_i^*)\, ]}$; 95% CI = $\mu \pm t(k-1, 0.975) \cdot SE\_HK$. This adjustment widens the interval to reflect the uncertainty in the estimation of between-study heterogeneity and is endorsed by the Cochrane Handbook (§10.10.4.4), reference [18] of the manuscript, where it is attributed to Hartung & Knapp (2001) and Sidik & Jonkman (2002).

### Interpretation

The pooled point estimates are directionally consistent across formulations and timepoints, but their statistical significance is sensitive to the conservative variance estimator appropriate for the small number of trials. Only DAS28-CRP change-from-baseline at 3 months ($I^2$ = 0%) remains significant under HKSJ. Relative to the DerSimonian–Laird intervals, the adjustment widened every pooled confidence interval except this one, for which the absence of between-study heterogeneity ($I^2$ = 0%) left it essentially unchanged.

The DAS28-ESR estimates, dominated by very high heterogeneity ($I^2 = 91\%$), support direction only, not a reliable estimate of effect.
