# Supplementary material for: The Impact of Periodontal Therapy on Disease Activity in Patients with Rheumatoid Arthritis and Concomitant Periodontitis: A Systematic Review and Meta-Analysis
Source: J Clin Med. 2026 Jun 30;15(13):5099. doi: 10.3390/jcm15135099 (PMC13362638; doi:10.3390/jcm15135099)
Supplement: Supplementary file 1 [file jcm-15-05099-s001.zip › Table_S1_Summary_of_findings.pdf]

Table S1. Summary of findings (GRADE certainty of evidence)

Summary of findings of GRADE certainty

Steps 1 and 2 of periodontal therapy compared to **no or delayed periodontal treatment, or oral hygiene instructions alone** for **adults with rheumatoid arthritis and concomitant periodontitis**

**Outcome**	**№ of participants (studies)**	**Relative effect (95% CI)**	**Anticipated absolute effect, MD (95% CI)**	**Certainty (GRADE)**[c]
DAS28-CRP, change from baseline (3 months)	189 (4 RCTs)	–	MD 0.55 lower (0.92 lower to 0.19 lower)	⊕⊕◯◯ **Low** [a,b]
DAS28-CRP, follow-up value (3 months)	189 (4 RCTs)	–	MD 0.84 lower (1.38 lower to 0.29 lower)	⊕⊕◯◯ **Low** [a,b]
DAS28-ESR, change from baseline (3 months)	211 (5 RCTs)	–	MD 1.27 lower (2.22 lower to 0.31 lower)	⊕◯◯◯ **Very low** [a,b,d]
DAS28-ESR, follow-up value (3 months)	211 (5 RCTs)	–	MD 0.89 lower (1.85 lower to 0.07 higher)	⊕◯◯◯ **Very low** [a,d,e]

CI, confidence interval; MD, mean difference; RCT, randomised controlled trial. Negative values favour periodontal therapy.

a No included trial was rated at low overall risk of bias (RoB-2); recurrent concerns in the randomisation process (D1) and open-label delivery of the intervention (D2); overall high risk of bias in Khare 2016 (DAS28-ESR) and in Thilagar 2022 and Nakajima 2025 (DAS28-CRP). Rated down one level.

b Few small trials; the 95% CI includes values below the EULAR threshold for a clinically perceptible improvement (0.6 units). Rated down one level.

c Funnel plots were not feasible (fewer than 10 trials per estimate); registered trials without published results were identified through trial registry records (indexed in CENTRAL), so publication bias is suspected but was not used as a separate reason for rating down.

d Substantial statistical heterogeneity (I^2 = 91%), only partially explained (post-hoc sensitivity analysis: I^2 = 0% after excluding trials with adjunctive antimicrobials). Rated down one level.

e The 95% CI crosses the line of no effect (it includes both an appreciable benefit and no effect). Rated down one level.

GRADE Working Group grades of evidence [33]

⊕⊕⊕⊕ **High certainty:** we are very confident that the true effect lies close to that of the estimate of the effect.

⊕⊕⊕◯ **Moderate certainty:** we are moderately confident in the effect estimate: the true effect is likely to be close to the estimate, but there is a possibility that it is substantially different.

⊕⊕◯◯ **Low certainty:** our confidence in the effect estimate is limited: the true effect may be substantially different from the estimate.

⊕◯◯◯ **Very low certainty:** we have very little confidence in the effect estimate: the true effect is likely to be substantially different from the estimate.
