# Supplementary material for: The Impact of Periodontal Therapy on Disease Activity in Patients with Rheumatoid Arthritis and Concomitant Periodontitis: A Systematic Review and Meta-Analysis
Source: J Clin Med. 2026 Jun 30;15(13):5099. doi: 10.3390/jcm15135099 (PMC13362638; doi:10.3390/jcm15135099)
Supplement: Supplementary file 1 [file jcm-15-05099-s001.zip › Table_S2_excluded_studies_JCM-2-2.pdf]

Table S2. Studies excluded at full-text assessment, with reasons for exclusion (n = 15).

Study	Reason for exclusion	Justification (verbatim citation)
Biyikoglu 2013	Wrong study design	Single-centre intervention study comparing treated RA + periodontitis with treated systemically healthy periodontitis, with no untreated RA arm: "This study was designed as a single-centered intervention study" [1].
Kurgan 2016	Wrong study design	Non-randomised before-and-after study in which the only untreated group comprised periodontally and systemically healthy controls: "This study was an observational clinical trial" [2].
Atarbashi-Moghadam 2018	Wrong study design	Allocation by patient preference (patients declining periodontal treatment formed the control group), not randomised: "Patients who refused periodontal treatment were classified as the control group (28 patients) and age- and sex-matched patients who were interested in periodontal treatment served as the case group (28 patients)" [3].
Cosgarea 2019	Wrong study design	Non-randomised intervention trial comparing treated RA + periodontitis with treated periodontitis without RA, with no untreated arm: "In this prospective, case-controlled, clinical intervention trial" [4].
Białowąs 2020	Wrong study design	Combined case–control and single-arm intervention study in a mixed RA/spondyloarthritis sample, with no untreated parallel arm: "The study was designed as both case–control and intervention study" [5].
Moura 2021 (Odontology)	Wrong study design	Non-randomised controlled trial in a convenience sample allocated by periodontitis/RA status, with no untreated parallel arm: "a controlled clinical trial … From a convenience sample, 107 individuals were considered eligible and consecutively allocated in four groups" [6].
Moura 2021 (Clin. Oral Investig.)	Wrong study design	Non-randomised trial in a convenience sample in which only the periodontitis + RA group received treatment: "The present non-randomized clinical trial" [7].
Posada-López 2022	Wrong study design	Quasi-experimental, non-randomised before-and-after study: "A quasi-experimental, prospective, non-randomized study" [8].
Popoca-Hernández 2024	Wrong study design	Quasi-experimental, single-arm before-and-after study in which each patient served as her own control: "In this quasi-experimental study, we evaluated 30 women … with periodontitis and RA" [9].
Buwembo 2020	Wrong comparator	Both arms received an antiseptic (cetrimide) mouthrinse, so the comparator was an active antimicrobial regimen rather than an untreated or oral-hygiene-only control: "Both study group subjects received a mouthwash, Citrollin (containing cetrimide and lidocaine HCl, from Pharco Pharmaceuticals, Alexandria, Egypt), and were advised to use 10 mL of the mouthwash twice a day after toothbrushing for 10 days after the OHI session" [10].
Silva DS 2025	Wrong comparator	Randomised delayed-start trial (n = 22), but all participants received treatment at baseline, leaving no parallel untreated arm: at V1 "all study participants were … submitted to supra and subgingival debridement", the deferred group's earlier assessment serving only "to act as a control group" [11].
Shimada 2016	Wrong intervention	Supragingival professional mechanical plaque removal only, without subgingival instrumentation: patients were "randomly assigned to receive periodontal treatment, including oral hygiene instruction and full-mouth supragingival scaling with ultrasonic instruments without local anesthesia" [12].

Study	Reason for exclusion	Justification (verbatim citation)
Ribeiro 2005	Wrong outcome	DAS28 was not measured; rheumatological status was assessed only by HAQ, RF and ESR: "Rheumatoid factor (RF), erythrocyte sedimentation rate (ESR) and drug therapy were assessed" [13].
Kaushal 2019	Wrong outcome	Rheumatoid disease activity was assessed with the Simplified Disease Activity Index rather than DAS28: "Rheumatologic clinical (simplified disease activity index)" [14].
Mariette 2020 (BHYRRA/ESPOIR)	Wrong population	General early-RA population (ESPOIR cohort) with periodontitis in only ≈56% at baseline, and an oral-hygiene intervention rather than steps 1 and 2 of periodontal therapy: "56% of patients had periodontal disease at baseline", the intervention comprising "general recommendations of good oral hygiene including teeth brushing, daily antiseptic mouthwash and twice a year scaling" [15].

Reasons are coded according to the PICOS eligibility criteria; category counts correspond to the PRISMA 2020 flow diagram (wrong study design, n = 9; wrong comparator, n = 2; wrong intervention, n = 1; wrong outcome, n = 2; wrong population, n = 1). Lopez-Oliva 2026 was identified as a companion report of the OPERA trial (de Pablo 2023) and treated as a secondary publication of an already-included study rather than as a separate exclusion. Full bibliographic references for the excluded studies are listed below, in order of appearance in the table.

Supplementary References

1. Bıyıkoğlu, B.; Buduneli, N.; Aksu, K.; Nalbantsoy, A.; Lappin, D.F.; Evrenosoğlu, E.; Kinane, D.F. Periodontal therapy in chronic periodontitis lowers gingival crevicular fluid interleukin-1beta and DAS28 in rheumatoid arthritis patients. *Rheumatol. Int.* **2013**, *33*, 2607–2616.
2. Kurgan, Ş.; Fentoğlu, Ö.; Önder, C.; Serdar, M.; Eser, F.; Tatakis, D.N.; Günhan, M. The effects of periodontal therapy on gingival crevicular fluid matrix metalloproteinase-8, interleukin-6 and prostaglandin E2 levels in patients with rheumatoid arthritis. *J. Periodontal Res.* **2016**, *51*, 586–595.
3. Atarbashi-Moghadam, F.; Rashidi Maybodi, F.; Dehghan, A.; Haerian Ardakani, A. Effect of non-surgical periodontal treatment on clinical signs of rheumatoid arthritis. *J. Adv. Periodontal Implant Dent.* **2018**, *10*, 13–17.
4. Cosgarea, R.; Tristiu, R.; Dumitru, R.B.; Arweiler, N.B.; Rednic, S.; Sirbu, C.I.; Lascu, L.; Sculean, A.; Eick, S. Effects of non-surgical periodontal therapy on periodontal laboratory and clinical data as well as on disease activity in patients with rheumatoid arthritis. *Clin. Oral Investig.* **2019**, *23*, 141–151.
5. Białowąs, K.; Radwan-Oczko, M.; Duś-Ilnicka, I.; Korman, L.; Świerkot, J. Periodontal disease and influence of periodontal treatment on disease activity in patients with rheumatoid arthritis and spondyloarthritis. *Rheumatol. Int.* **2020**, *40*, 455–463.
6. Moura, M.F.; Cota, L.O.M.; Silva, T.A.; Cortelli, S.C.; Ferreira, G.A.; López, M.M.; Cortelli, J.R.; Costa, F.O. Clinical and microbiological effects of non-surgical periodontal treatment in individuals with rheumatoid arthritis: a controlled clinical trial. *Odontology* **2021**, *109*, 484–493.
7. Moura, M.F.; Silva, T.A.; Cota, L.O.M.; Oliveira, S.R.; Cunha, F.Q.; Ferreira, G.A.; Cortelli, J.R.; Cortelli, S.C.; Costa, F.O. Nonsurgical periodontal therapy decreases the severity of rheumatoid arthritis and the plasmatic and salivary levels of RANKL and Survivin: a short-term clinical study. *Clin. Oral Investig.* **2021**, *25*, 6643–6652.
8. Posada-López, A.; Botero, J.E.; Pineda-Tamayo, R.A.; Agudelo-Suárez, A.A. The effect of periodontal treatment on clinical and biological indicators, quality of life, and oral health in rheumatoid arthritis patients: a quasi-experimental study. *Int. J. Environ. Res. Public Health* **2022**, *19*, 1789.
9. Popoca-Hernández, E.A.; Martínez-Martínez, R.E.; González-Amaro, R.F.; Niño-Moreno, P.D.; Ayala-Herrera, J.L.; Jerezano-Domínguez, A.V.; Espinosa-Cristóbal, L.F.; Márquez-Corona, M.D.; Santillana, I.A.E.D.; Medina-Solís, C.E. Impact of non-surgical periodontal treatment on the concentration and level of MRP-8/14 (calprotectin) as an inflammatory biomarker in women with periodontitis and rheumatoid arthritis: a quasi-experimental study. *Diseases* **2024**, *12*, 12.

10. Buwembo, W.; Munabi, I.G.; Kaddumukasa, M.; Kiryowa, H.; Mbabali, M.; Nankya, E.; Johnson, W.E.; Okello, E.; Sewankambo, N.K. Non-surgical oral hygiene interventions on disease activity of rheumatoid arthritis patients with periodontitis: a randomized controlled trial. *J. Dent. Res. Dent. Clin. Dent. Prospects* **2020**, *14*, 26–36.
11. Silva, D.S.; de Vries, C.; Rovisco, J.; Serra, S.; Kaminska, M.; Mydel, P.; Lundberg, K.; da Silva, J.A.P.; Baptista, I.P. The impact of periodontitis and periodontal treatment on rheumatoid arthritis outcomes: an exploratory clinical trial. *Rheumatology* **2025**, *64*, 1679–1688.
12. Shimada, A.; Kobayashi, T.; Ito, S.; Okada, M.; Murasawa, A.; Nakazono, K.; Yoshie, H. Expression of anti-*Porphyromonas gingivalis* peptidylarginine deiminase immunoglobulin G and peptidylarginine deiminase-4 in patients with rheumatoid arthritis and periodontitis. *J. Periodontal Res.* **2016**, *51*, 103–111.
13. Ribeiro, J.; Leão, A.; Novaes, A.B. Periodontal infection as a possible severity factor for rheumatoid arthritis. *J. Clin. Periodontol.* **2005**, *32*, 412–416.
14. Kaushal, S.; Singh, A.K.; Lal, N.; Das, S.K.; Mahdi, A.A. Effect of periodontal therapy on disease activity in patients of rheumatoid arthritis with chronic periodontitis. *J. Oral Biol. Craniofac. Res.* **2019**, *9*, 128–132.
15. Mariette, X.; Perrodeau, E.; Verner, C.; Struillou, X.; Picard, N.; Schaeverbeke, T.; Constantin, A.; Ravaud, P.; Bouchard, P. Role of good oral hygiene on clinical evolution of rheumatoid arthritis: a randomized study nested in the ESPOIR cohort. *Rheumatology* **2020**, *59*, 988–996.
